# TURis plasma vaporization in non–muscle invasive bladder cancer–the first Romanian experience with a new technique

**Published:** 2010-02-25

**Authors:** B Geavlete, M Jecu, R Mulţescu, D Georgescu, M Drăguţescu, P Geavlete

**Affiliations:** ‘St. John’ Clinical Emergency Hospital, Department of Urology, Bucharest Romania

**Keywords:** non–muscle invasive bladder tumors, transurethral resection in saline–plasma vaporization of bladder tumors, 3 months recurrence rate

## Abstract

Introduction: The development of bipolar resection using saline irrigation provided significant improvements in NMIBT treatment. The aim of our study was to evaluate the efficacy and safety of a newly introduced endoscopic technique, the bipolar transurethral resection in saline–plasma vaporization of bladder tumors (TURis–PVBT).

Materials and Methods: Between May and October 2009, 57 consecutive patients presenting papillary bladder tumors larger than 1 cm underwent TURis–PVBT and a 3 months follow–up. Initial tumor biopsy, followed by plasma vaporization of the tumor and biopsies of the tumoral bed were performed in all cases. Complementary treatment was applied according to risk indications. The follow–up protocol included abdominal ultrasonography, cytology and cystoscopy at 3 months.

Results: TURis–PVBT was successfully performed in all cases. Multiple tumors were found in 45.6% of the cases and 50.9% of the patients presented tumors larger than 3 cm. The mean tumoral volume was of 11 ml. The mean operative time was of 17 minutes, the mean hemoglobin decrease was of 0.4 g/dl, the mean catheterization period was of 2.5 days and the mean hospital stay was of 3.5 days. The pathological exam diagnosed 57.9% pTa cases, 31.6% pT1 cases and 10.5% pT2 cases. No tumoral base biopsies were positive for malignancy. The recurrence rate at 3 months for the 51 NMIBT patients was of 15.7%. Orthotopic recurrent tumors were encountered in 5.9% of the cases.

Conclusions: TURis–PVBT seems to represent a promising endoscopic treatment alternative for NMIBT patients, with good efficacy, reduced morbidity, fast postoperative recovery and satisfactory follow–up parameters.

## Introduction

The endoscopic treatment of NMIBT patients represented a challenge for the urologist since the very beginning. From the first resectoscope invented by Stern in New York in 1926 [1] to the modern instruments using bipolar energy, TURBT faced many shortcomings, as well as a significant rate of intra–and postoperative complications.

According to the EAU Guidelines 2009, TURBT aiming to achieve complete macroscopic eradication including a part of the underlying muscle represents the standard therapy for Ta and T1 papillary bladder tumors. The goal of TURBT is to make the correct diagnosis and to remove all the visible lesions [2].

However, bladder tumors characterized by specific features, such as their location in places that are difficult to be accessed (bladder dome, anterior bladder wall) or subject to obturator nerve stimulation (lateral bladder walls), as well as their size (larger than 3 cm), imposed the search for improvement in endoscopic surgical approach. 

On the other hand, the significant complications of standard monopolar resection, consisting of bladder wall perforation, intra– and postoperative bleeding (eventually imposing blood transfusions or reintervention), urinary retention by blood clots, obturator nerve stimulation, tumoral spilling and urethral strictures also demanded the search for new alternatives.

The bipolar transurethral resection in saline (TURis) proved to offer the patients the same results as monopolar technology, thus guaranteeing maximum safety without increasing the incidence of urethral strictures [3].

TURis plasma vaporization was recently introduced in the armamentarium treatment for benign prostatic hyperplasia (BPH) and described as a safe and effective treatment option for patients with low urinary tract symptoms due to bladder outlet obstruction [4].

Following the tradition of promoting new techniques in endourology, the Department of Urology of ‘St. John’ Clinical Emergency Hospital introduced TURis plasma vaporization as an absolute national premiere in Romania, in May 2009. 

It was initially performed for BPH patients, but as soon as we realized the potential benefits of tumoral tissue plasma vaporization, we started applying this technique to papillary bladder tumors as well. With this regard, we may proudly state that the Romanian experience of ‘St. John’ Department of Urology is among the very few existing at this moment, in the world.

In this study, we aimed at evaluating the efficiency, safety and short-term postoperative results of this new endoscopic technique.

## Material and methods

Between May and October 2009, 57 consecutive patients with papillary bladder tumors underwent TURis–PVBT and a 3 months follow–up. 

The Olympus SurgMaster UES–40 bipolar generator, the special ‘mushroom’ type vapo– resection electrode and the continuous saline flow irrigation were used in all cases.

All patients underwent a standard investigation protocol, which included general clinical examination, blood tests, urine culture, abdominal ultrasonography, intravenous pyelography and eventually CT–scan. 

All the procedures were carried out under spinal anesthesia by a single surgeon. Plasma vaporization was performed only for papillary tumors larger than 1 cm in diameter. 

**Fig 1:**
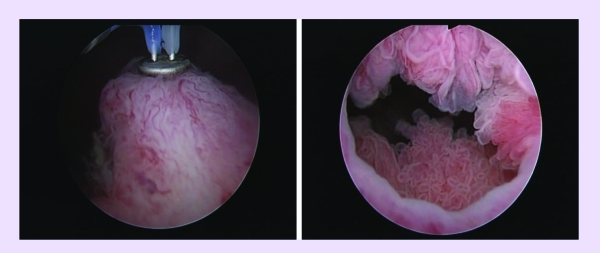
Large bladder tumors before plasma vaporization

The procedure started with a comprehensive cystoscopy, determining the presence, size and location of all existing tumors (Fig 1).

**Fig 2:**
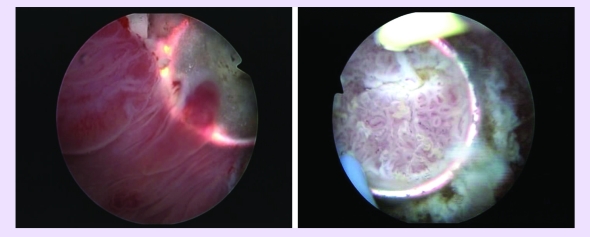
Bipolar resection of several tumoral tissue specimens

**Fig 3:**
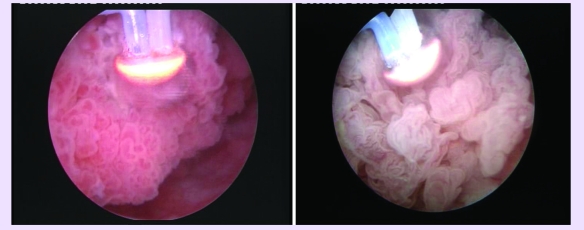
Initial aspects at the beginning of TURis–PVBT

The next step consisted of bipolar resection of several tumoral tissue specimens for the pathological analysis (Fig 2), and was followed by the actual plasma vaporization (Fig 3).

**Fig 4:**
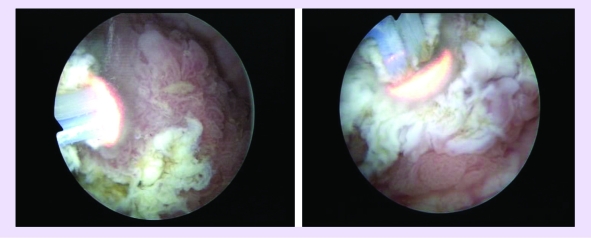
Tumor vaporization by the plasma corona on the surface of the ‘mushroom’ type electrode

The spherical shaped new type of electrode displaying a plasma corona on its surface was gradually moved in direct contact with the tumoral tissue (the ‘hovering’ technique), thus producing a virtually blood–less vaporization at 280 W (Fig 4).

Coagulation of any hemorrhagic sources was practically concomitant, while larger vessels' hemostasis was achieved by reducing the power of the generator to 120–140 W.

**Fig 5:**
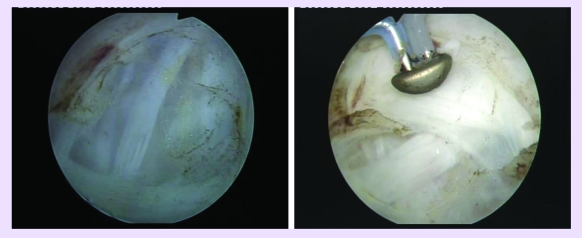
Postoperative images showing the clean muscular layer of the bladder wall

Tumor vaporization enabled the surgeon to clearly visualize the muscular layer of the bladder wall (Fig 5).

**Fig 6:**
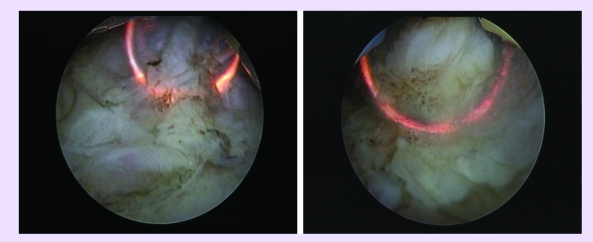
Bipolar resection of the center and margins of the tumoral bed

**Fig 7:**
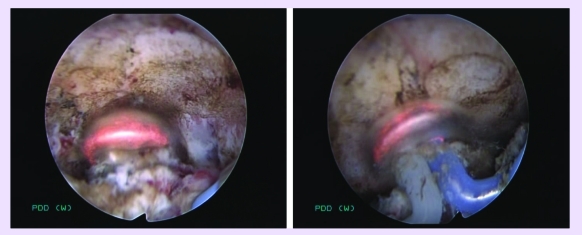
Coagulation of the tumoral bed and margins of the resection area

Consequently, bipolar resection of the center and margins of the tumoral bed was performed for the pathological confirmation of the complete tumor removal (Fig 6). The coagulation of the tumoral bed and margins of the resection area also using the ‘mushroom’ electrode, took place at the end of the procedure (Fig 7). 

A single mitomycin–C instillation was performed during the first 6 hours after the procedure in all cases. A complementary intravesical treatment consisting of mitomycin–C or BCG instillations was applied in NMIBT patients according to their risk group. The muscle–invasive bladder cancer cases were excluded from the study and scheduled for radical cystectomy. 

The follow–up was performed 3 months after surgery for all NMIBT cases. The follow–up protocol included abdominal ultrasonography, urinary cytology and cystoscopy.

## Results

TURis–PVBT was successfully performed in all cases. The mean patients' age was of 61 years old (between 36 and 85 years old). The mean tumoral volume was of 11 ml (between 4 and 35 ml). The proportions of cases presenting solitary tumors smaller and respectively larger than 3 cm were of 21.1% and 33.3%, while for multiple tumors, these percentages were of 28.1% and 17.5%, respectively. 

Consequently, tumors larger than 3 cm were encountered in 50.9% of the cases and multiple tumors were found in 45.6% of the patients. Recurrent bladder cancer was diagnosed in 22.8% of the patients, while 77.2% of the cases presented primary tumors.

The mean operative time was of 17 minutes (ranging from 6 to 35 minutes) and the mean postoperative hemoglobin decrease was of 0.4 g/dl (ranging from 0.2 and 0.9 g/dl). An immediate postoperative instillation of mytomicin C was applied in all patients.

The mean catheterization period was of 2.5 days (between 2 and 3.5 days) and the mean hospital stay was of 3.5 days (between 2.5 and 4.5 days). 

Initial tumoral biopsy, followed by plasma vaporization of the tumor and biopsy of the tumoral bed were successfully completed for every patient.

We encountered no case of bladder wall perforation, persistent intraoperative bleeding or postoperative urinary retention by blood clots. None of the patients required blood transfusion or reintervention. There was a single case of obturator nerve stimulation (1.7') and another (1.7') of prolonged postoperative mild hematuria, successfully treated with conservative means. 

The pathological exam diagnosed 57.9% pTa cases, 31.6% pT1 cases and 10.5% pT2 cases. All resected specimens were valid for pathological analysis. No tumoral base biopsies were positive for malignancy and they included muscular fibers in all cases. The six muscle–invasive bladder cancer cases underwent radical cystectomy.

The recurrence rate at 3 months for the 51 NMIBT patients was of 15.7%. In this series, 21.6% of the patients initially had single tumors smaller than 3 cm, 33.3% single tumors larger than 3 cm, 27.5% multiple tumors smaller than 3 cm and 17.6% multiple tumors larger than 3 cm). 

Orthotopic recurrences alone were encountered in 3.9% of the cases, 9.8% of the patients only had heterotopic tumors and 2% (1 patient) had both ortho– and heterotopic recurrences. The recurrence rate was of 9.1% for patients with initial single tumor smaller than 3 cm and of 17.5% in cases of single tumors larger than 3 cm or of multiple tumors. 

## Discussion

TURi–PVBT is a new procedure, at the very beginning as part of the treatment armamentarium for non–muscle invasive bladder cancer. While gaining an increasing acknowledgement as a reliable therapeutic alternative for BPH [4], the use of this method in NMIBT patients has not yet been evaluated in clinical trials. Therefore, one of the most important features of the present study is related to its pioneering character.

From the technical point of view, TURis–PVBT ensured a high quality tumor ablation with excellent visibility due to minimal bleeding. The advantages of the procedure mainly consisted of: reduced stimulation of the obturator nerve, lack of bladder perforation and virtually no major postoperative bleeding.

Subjectively, this type of vaporization did not alter the visual characteristics of the anatomical layers, enabling the surgeon to differentiate between the tumoral tissue, the muscular fibers of the bladder wall, as well as the clear boundaries of the operating area, with increased accuracy. Due to the lack of bleeding, visibility remained excellent throughout the procedure. The vaporization area emphasized a remarkably smooth surface and sharp margins, with no irregularities or debris. 

As some stages of the conventional TURBT were significantly reduced (concomitant vaporization and hemostasis, rapid evacuation of the few resected tissue fragments), the actual vaporization occupied the great majority of the operating time and increased the efficiency of TURis–PVBT.

The comparison of this method with the literature data seems to confirm the potential advantages provided by TURis–PVBT. A study performed by Puppo et al. concerning bipolar TURBT, involving a number of 480 patients with similar parameters and using the same TURis generator as the one in our study, determined a mean operating time of 27 minutes, an obturator nerve stimulation rate of 2%, a mean hemoglobin decrease of 0.7 g/dl, a transfusion rate of 0.8%, a blood clot retention rate of 2% and a mean catheterization period of 3 days [3].

Although rather small, our series seems to emphasize improved results in all regards: a mean operative time of 17 minutes, a 1.7% rate of obturator nerve stimulation, a mean postoperative hemoglobin decrease of 0.4 g/dl, a mean catheterization period of 2.5 days and no cases of blood transfusion or blood clot retention.

The standard TURBT is still marked by a significant number of complications, which new treatment alternatives aim to avoid. The extensive study by Collado et al. on 2821 patients assessed the most common complications related to monopolar TURBT and determined a bleeding rate of 2.8%, a perforation rate of 1.3%, a re–intervention rate of 2.7% and a blood transfusion rate of 3.4%. [5] 

The fact that none of these complications occurred in our study group can be considered quite promising, as TURis–PVBT seems to optimize efficacy and to increase safety during the endoscopic approach of bladder tumors. The catheterization period was also longer for monopolar TURBT (5 days versus 2.5 days in our series).

An important issue is represented by the difficulties concerning the resection of large bladder tumors, which many times imply important bleeding rates, prolonged resection time, poor visibility as well as an increased rate of complications. For example, Collado et al. reported a 6.7% rate of complications in cases of tumors larger than 3 cm. [5]

Maybe one of the most important benefits of TURis-PVBT is represented by the rapid vaporization of large quantities of tumoral tissue with minimal blood loss. We were able to successfully treat the 29 cases of this kind from our series, without complications.

On the other hand, the resected specimens were sufficient for the pathological analysis and included muscular layer for every tumor, so that stage and grading were clearly determined in all cases.

There are various results mentioned in the literature data concerning the recurrence rate at the 3 months follow–up cystoscopy. A study by Guney et al. on 641 patients during a period of 10 years determined a recurrence rate of 21% at 3 months. [6] On the other hand, the combined analysis of seven EORTC studies performed by Brausi et al. on 2410 NMIBT patients determined a 3 months recurrence rate of 13.1% [7]. 

Consequently, it would only be fair to say that the 15.7% 3 months recurrence rate in our series appears to confirm the fact that TURis–PVBT is a successful approach in terms of oncological safety as well, with a short–term recurrence rate that matches the one of monopolar TURBT. 

According to the same meta–analysis by Brausi et al., the rate of orthotopic recurrences was of 8.7%, while 3.2% of the patients presented heterotopic recurrences and 3.7% of the cases were diagnosed with both ortho– and heterotopic recurrent tumors during the 3 months cystoscopy follow–up. [7] 

In our series, we recorded a 3.9% rate of orthotopic recurrences, a 9.8% rate of heterotopic tumors and a single case (2%) which had both ortho– and heterotopic tumors. The practically inversed proportions of ortho– and heterotopic recurrences between the two studies seem to suggest the superior rate of initial complete removal of bladder tumors achieved by TURis–PVBT.

In a study published by Divrik et al. with regard to short–term recurrences after monopolar TURBT, the recurrence rate in patients initially diagnosed with a single tumor smaller than 3 cm was of 20%, while in cases of single tumors larger than 3 cm or of multiple tumors, the respective rate was of 37%. [8] 

In our series, these recurrence rates were of 9.1% and 17.5%, respectively. Consequently, TURis–PVBT appears to display remarkable efficacy in terms of oncological results as well in cases of large or multiple bladder tumors.

## Conclusions

We may conclude that TURis–PVBT seems to represent a promising endoscopic treatment alternative for NMIBT patients, with good efficacy, reduced morbidity, fast postoperative recovery and satisfactory follow– up parameters by comparison to bipolar as well as to standard monopolar TURBT. 

Longer follow–up periods and trials that are more extensive will be required in order to establish the long–term advantages and general viability of the method as a therapeutic approach in bladder cancer. 

However, the remarkably efficient tumoral tissue vaporization, excellent visibility, reduced intra– and postoperative bleeding, short period of catheterization and hospital stay, lack of complications and good oncological results represent reliable arguments in favor of this new procedure.
